# Supermode-density-wave-polariton condensation with a Bose–Einstein condensate in a multimode cavity

**DOI:** 10.1038/ncomms14386

**Published:** 2017-02-17

**Authors:** Alicia J. Kollár, Alexander T. Papageorge, Varun D. Vaidya, Yudan Guo, Jonathan Keeling, Benjamin L. Lev

**Affiliations:** 1 Department of Applied Physics, Stanford University, MC 305, Stanford, California 94305, USA; 2 E.L. Ginzton Laboratory, Stanford University, MC 305, Stanford, California 94305-4088, USA; 3 Department of Physics, Stanford University, Stanford, MC 305, California 94305, USA; 4 SUPA, School of Physics and Astronomy, University of St Andrews, St Andrews KY16 9SS, UK

## Abstract

Phase transitions, where observable properties of a many-body system change discontinuously, can occur in both open and closed systems. By placing cold atoms in optical cavities and inducing strong coupling between light and excitations of the atoms, one can experimentally study phase transitions of open quantum systems. Here we observe and study a non-equilibrium phase transition, the condensation of supermode-density-wave polaritons. These polaritons are formed from a superposition of cavity photon eigenmodes (a supermode), coupled to atomic density waves of a quantum gas. As the cavity supports multiple photon spatial modes and because the light–matter coupling can be comparable to the energy splitting of these modes, the composition of the supermode polariton is changed by the light–matter coupling on condensation. By demonstrating the ability to observe and understand density-wave-polariton condensation in the few-mode-degenerate cavity regime, our results show the potential to study similar questions in fully multimode cavities.

## Abstract (web summary)

When a single mode optical cavity is coupled to a Bose-Einstein condensate, one usually observes a single mode of light when strongly pumped. Here the authors observe a supermode in the output of a multimode cavity and relate this to a signature of a nonequilibrium condensation phase transition.

Ultracold atoms have provided an exemplary model system to demonstrate the physics of closed-system, equilibrium phase transitions, confirming many theoretical models and results^1^. A striking manifestation of the role quantum mechanics can play in the physics of equilibrium phase transitions is the well-known Bose–Einstein condensation (BEC) of bosonic particles at low temperatures into a single, macroscopically populated quantum wave. Our understanding of dissipative phase transitions in quantum systems is less developed and experiments that probe this physics even less so. For example, condensation in quantum systems out of thermal equilibrium is far less understood than in equilibrium^2^,^3^, yet becoming experimentally relevant, especially via the study of polariton condensates: when matter couples strongly to light, new collective modes called polaritons arise. Condensation of these quasiparticles has been actively studied in the form of exciton polaritons^4^,^5^,^6^,^7^,^8^,^9^,^10^,^11^.

With only a single mode of light coupled to the BEC, self-organization has been observed, but is uniquely defined by cavity geometry alone and is best described as a density-wave (DW) polariton condensate^12^,^13^,^14^. In contrast, for exciton polaritons, complex pattern formation^8^,^9^,^10^ has been seen, arising from the existence of many nearly degenerate modes in planar microcavities. In addition, related physics has also been observed in systems in which no DW-polariton condensation occurs: for example, superradiant emission using hyperfine states^15^, supermode emission without self-organization^16^ and self-organization without a cavity^17^. The Bose–Hubbard model with infinite-range interactions has been studied^18^,^19^. We note that condensation of DW polaritons is quite distinct from lasing: standard lasing requires inversion of the gain medium, is driven by an incoherent pump and is generally incompatible with strong light–matter coupling, whereas the transition we study differs on all of these points.

We report a step towards the observation of an unusual form of non-equilibrium condensation, a ‘supermode-DW-polariton' condensate. Here, supermode refers to the eigenmode built as a superposition of the bare cavity modes^20^. The supermode-polariton dressed state is dependent on the pump-cavity detuning Δ_c_ and on the overlap of the bare-cavity modes with the BEC position and shape. The matter component is an atomic DW excitation rather than the electronic excitation of exciton-polariton condensates. It is important to distinguish the condensation of supermode DW polaritons from condensation of the atoms; for example, self-consistent formation of atomic DWs and cavity light can be studied with thermal atoms^21^,^22^ and with BECs^12^. However, some of the signatures we demonstrate, such as the atomic structure factor, are observable only with BECs of atoms in multimode cavities (see Methods for further discussion). By embedding the exquisite control available for ultracold atoms within multimode quantum-optical systems, our experiment opens avenues for experimentally studying quantum fluctuation-driven transitions and quantum criticality^2^,^23^,^24^,^25^. In such a system, one can expect physics beyond mean-field theories such as a quantum Brazovskii transition or a short-range spin glass^23^,^26^. Thus, these systems will provide experimental access to non-trivial phase transitions in driven dissipative quantum systems^2^,^3^,^23^,^24^,^27^ and enabling the studies of exotic non-equilibrium spin glasses and neuromorphic computation^26^,^28^.

## Results

### Supermode DW polaritons

**Figure 1 f1:**
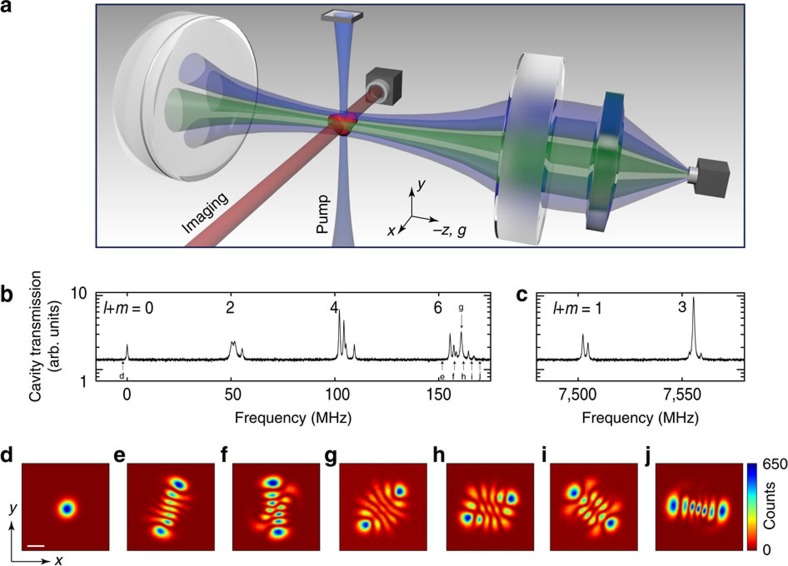
Experimental apparatus and cavity spectrum and superradiant emission. (**a**) The ^87^Rb BEC is trapped at the centre of the cavity and at the focus of the standing wave transverse pump far detuned from electronic transitions. (ODT lasers not shown.) Detection channels include absorption imaging of atomic density and detection of cavity emission using either a single-photon counter, a photodetector or an electron-multiplying CCD camera (shown). (**b**,**c**) Cavity transmission showing near-degenerate bare-cavity mode families (that is, modes with *l*+*m*=const.) versus frequency. These are measured with a longitudinal probe (not shown) in the near-confocal regime for (**b**) even modes and (**c**) odd modes. (**d**) Superradiant emission in the transverse electromagnetic *l*=*m*=0 (TEM_00_) mode when pumped above DW polariton condensation threshold at position *d* in **b**. (**e**–**j**) Superradiant photonic components of various supermode-DW-polariton condensates of the *l*+*m*=6 family. The colourbar scale for cavity emission in **d**–**j** is to the right of **j**. For all **d**–**j**, the axes and scale are indicated with the labelled arrows and the white bar as in **d**, where the bar equals twice the TEM_00_ optical waist 2*w*_0_=70 μm at the BEC.

In the work reported here, we see how with a few modes coupled to the BEC (see Fig. 1b,c) the supermode-DW-polariton condensate arises in a regime intermediate between BECs coupled to a single-mode cavity and those coupled to a confocal or concentric cavity supporting many hundreds of degenerate modes. When the atoms are pumped with a laser orthogonal to the cavity axis (see Fig. 1a), the normal modes of the atom-cavity system evolve to become supermode DW polaritons, new superpositions of supermodes mixed by the DW fluctuations of the atoms. Above a critical pump threshold, we see defining characteristics of supermode-DW-polariton condensates, heralded by three observables as follows: first, superradiant emission of light from the cavity with the spatial pattern of one of these new supermodes; second, *Z*_2_ symmetry breaking of the phase of the cavity field, locking to either 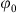 or 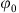
+*π* with respect to the pump phase; and third, organization of the BEC wavefunction into one of two checkerboard lattice configurations—each corresponding to a specific phase of the cavity field. Such observables have been seen in single-mode cavities^12^. In addition, we see how non-trivial transverse spatial structure of the supermode can result in lattice defects (matter–wave phase slips): these are seen in the structure factor in an atomic time-of-flight measurement. Observations of all these defining characteristics are presented.

The near-confocal optical cavity employed here supports families of optical modes that each lie within a small frequency bandwidth, as shown in Fig. 1b,c (see Methods). One can observe the superradiant emission of various supermode-DW-polariton condensates by pumping at different Δ_c_ tuned near or within a mode family, as can be seen in Fig. 1e,j. This is in contrast to the DW-polariton condensate of a single-mode cavity such as shown in Fig. 1d: This is not a supermode no matter the detuning Δ_c_ near this isolated Gaussian mode. The supermodes in Fig. 1e–j, [Fig f1] differ from ideal Hermite–Gaussian modes due to three factors as follows: first, the bare cavity modes are themselves mixtures of ideal Hermite–Gaussian modes (due to mirror aberrations mixing modes when there is spectral overlap of modes near degeneracy)^29^; second, these bare cavity modes are mixed by the dielectric atomic medium to form supermode polaritons; and third, these dressed states are remixed by the emergent DW to form new supermodes above the polariton condensation threshold.

### Decomposition of photonic components

**Figure 2:**
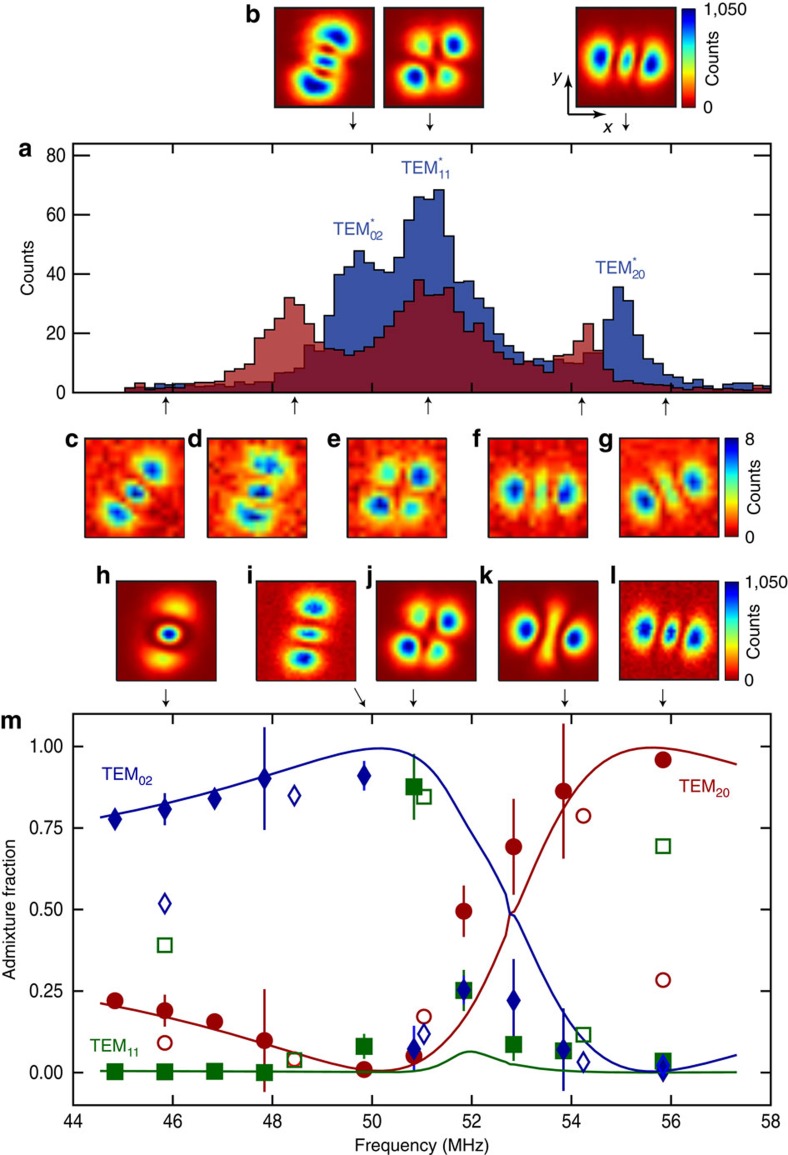
Photonic composition. (**a**) Mode spectrum without atoms (blue) and with atoms (red) of the *l*+*m*=2 family versus frequency measured on a single-photon counter with a longitudinal probe. Frequency scale relative to TEM_00_ mode in Fig. 1b. The three supermode peaks of the spectrum with atoms exhibit a red dispersive shift (see Methods). Images of cavity transmission at the three bare cavity mode peaks (no atoms present) are shown in **b**. The asterisks in the mode labels 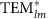 indicate that these bare cavity modes are not quite ideal TEM modes due to small intrafamily mode mixing by mirror aberrations. The 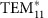 is unshifted due to poor overlap with centrally trapped BEC. (**c**–**g**) Images of the photonic component of the supermode-polariton dressed states and (**h**–**l**) supermode-DW-polariton condensate (pump above threshold) versus transverse-probe frequency. The photonic composition of the near-resonance supermode DW polaritons **i**–**k** corresponds closely to that of the resonant supermodes **e**,**f**. However, the supermode-DW condensates away from resonance, **h** and **l**, significantly differ from the supermodes away from resonance, **c** and **g**, demonstrating the remixing of supermodes due to DW–supermode coupling above threshold. (**m**) Admixture versus frequency of ideal cavity modes in the supermode-DW-polariton condensates (solid points) and the supermode polaritons (open points). Error bars represent 1 s.e. Solid lines are predicted supermode-DW-polariton condensate compositions. The colourbar for cavity emission in **b** is shown to the right of the rightmost subfigure. The colourbar for **c**–**i** is to the right of **g**,**i**. For all **b**–**l**, the axes and scale are indicated with the labelled arrows and the white bar as in **b**, where the bar equals twice the TEM_00_ optical waist 2*w*_0_=70 μm at the BEC.

Figure 2 illustrates how remixed photonic components of the supermode-DW-polariton condensate can differ from the supermode-polariton dressed states. The three bare cavity modes of the *l*+*m*=2 family are mixed by a BEC at the cavity centre to produce the three supermode peaks. The photonic component, shown in Fig. 2h–l, of the supermode-DW-polariton condensates can differ from that of the supermode polaritons in Fig. 2c–g due to new supermode mixing by the macroscopically populated atomic DW above threshold. This is most pronounced away from resonance: see, for example, how Fig. 2c,h differ; the supermode-DW-polariton condensate at 46 MHz (Fig. 2h) is ∼81% TEM_02_ and 19% TEM_20_, whereas the associated below-threshold supermode polariton (Fig. 2c) is ∼52% TEM_02_, 9% TEM_20_ and 39% TEM_11_. The suppression of the TEM_11_ component above threshold can be understood as resulting from its poor overlap with the BEC and thus weaker mixing with the DW mode. A similar remixing of supermodes occurs on the blue-detuned side at 56 MHz.

The components closely follow the theory prediction based on a linear stability analysis for mode content at condensation threshold, except near the 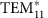 mode. We believe this discrepancy is due to dynamical effects not captured by this static stability analysis. See Fig. 8 in Methods for discussion.

### Position dependence

**Figure 3:**
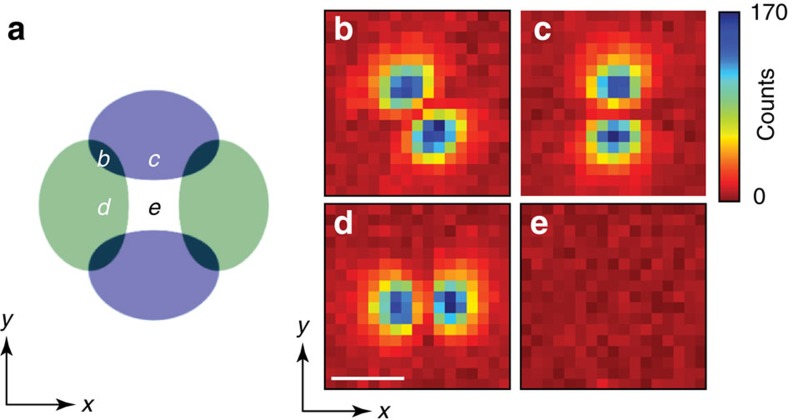
Dependence on BEC position. (**a**) Schematic of the cavity transverse plane with the bare cavity TEM_01_ and TEM_10_ modes in blue and green, respectively. Letters indicate positions of the ODT confining the BEC, whose width is much smaller than cavity mode waist (see Methods). (**b**–**e**) Moving the BEC from positions **b** through **d** changes the supermode-DW-polariton condensate composition, as may be seen by the superradiant emission in the correspondingly numbered panels. Images taken for same above-threshold pump power and detuning Δ_c_=−30 MHz. The astigmatic splitting of the two modes is much smaller, 2.4 MHz. Position **e** coincides with the *l*+*m*=1 family node: poor overlap lowers the coupling and threshold is not reached for this power. The colourbar for cavity emission in **b**–**e** is to the right of **c**. For all **b**–**e**, the axes and scale are indicated with the labeled arrows and the white bar as in **d**, where the bar equals twice the TEM_00_ optical waist 2*w*_0_=70 μm at the BEC.

For the limit we consider, where the atomic cloud is smaller than the beam waist, the position of the BEC with respect to the bare-cavity modes strongly affects the photonic mode composition of the supermode-DW-polariton condensate. That is, the near-degeneracy of the bare cavity modes means that the particular supermode selected is determined by overlap with the cloud. This is easily observed by moving the BEC within the transverse plane of the cavity with the pump tuned near the *l*+*m*=1 family (see Fig. 3). We pump the system with the BEC trapped by the optical dipole trap (ODT) at each of the four intracavity positions illustrated in Fig. 3a. With the BEC trapped near either the antinode of the TEM_10_ or the TEM_01_ mode, we observe superradiant emission with a spatial pattern nearly identical to these bare-cavity modes, as shown in Fig. 3c,d. However, a BEC at the intersection between the two modes' antinodal lobes (Fig. 3b) yields an emitted spatial pattern at 45° to the bare cavity mode axes. The threshold for organization is the same for a BEC at a bare-cavity antinode or between the antinodes, demonstrating that the BEC has mixed these bare modes equally and has created a basis for supermode DW polaritons that are rotated 45° from the original *l*+*m*=1 family eigenbasis; see Methods.

### Structure factor

**Figure 4:**
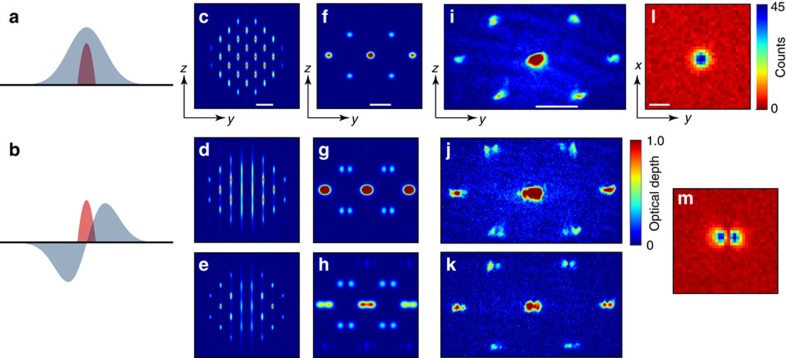
Structure factor measurement. (**a**–**h**) Simulation of BEC organization (see Methods). Sketch of BEC (red) at the centre of (**a**) TEM_00_ or (**b**) TEM_01_ modes (blue). Calculated atomic density distribution shown *in situ* in **c**–**e** and after time-of-flight expansion in **f**–**h**. (**a**) A BEC coupled to the antinode of a field organizes into a defectless checkerboard lattice above threshold (shown in **c**), exhibiting a featureless structure factor in the experimentally observed Bragg peaks shown in the time-of-flight image data in **i** (similar to the theory calculation in **f**). The associated cavity emission in the TEM_00_ mode is shown in **l**. (These manifestations of DW-polariton condensation are not observable in thermal gases.) (**b**) By contrast, BECs located at a field node—here that of a TEM_01_ mode—self-organize into a checkerboard lattice with a line defect at the optical node interface, shown in **d**. (**g**) This introduces a non-trivial atomic structure factor manifest as a node in the first-order Bragg peaks of the momentum distribution. (**j**) Indeed, these nodes are clear in the time-of-flight atomic density image data for condensation into the primarily TEM_01_ mode, seen in the cavity emission data of **m**. The atomic density image data in **k** shows that the zero-order peak can develop structure if the system is driven with a higher pump power, as simulated in **e**,**h** for *in-situ* and time-of-flight, respectively. This increases the coupling nonlinearity, deepening the optical potential and changing the BEC wavefunction via multiple Bragg scattering events. The colourbar for **c**–**k** is shown to the right of **j**. The colourbar for cavity emission in **l**,**m** is to the right of **l**. For all **c**–**k**, the axes are indicated with labelled arrows in **c**,**f**,**i** for the atomic density and in **l** for cavity emission. The white scale bars equal **c**, *λ*=780 nm; **f**,**i**, the recoil wavevector *k*_r_=2*π*/*λ*; and **l**, twice the TEM_00_ optical waist 2*w*_0_=70 μm at the BEC.

To complete the description of this non-equilibrium condensate, we report that observations of the momentum distribution in time-of-flight reveal the influence of the supermode structure on the matter wave component of the polariton condensate. For single-mode cavities pumped near the TEM_00_ mode, the atoms organize in one of two possible checkerboard patterns. However, organization in cavities supporting higher-order modes is more complicated. As illustrated in Fig. 4a versus Fig. 4b when the BEC overlaps with a node of the cavity mode, the effective DW—cavity mode coupling changes sign across the node, because the DW couples to the interference between pump field and cavity mode. This results in an organized state with a plane defect in the checkerboard lattice: that is, a *π* phase slip, and we confirm this configuration to be the optimal organized state via open-system simulations of the pumped BEC-cavity system. See Fig. 4d and Methods.

**Figure 5:**
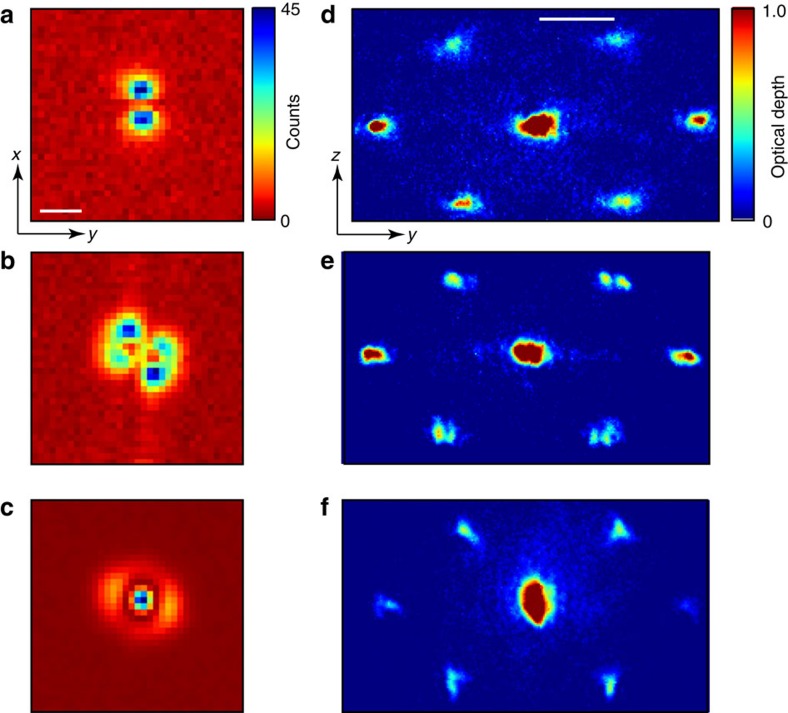
Structure factor measurement in higher-order families. (**a**–**c**) Images of cavity emission of photonic components of a supermode-DW-polariton condensate for modes (**a**) TEM_10_; (**b**) 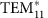, which is TEM_11_ with only a few per cent admixture of TEM_02_ and TEM_20_; and (**c**) TEM_*α*02+*β*20_, with *α*≈81% and *β*≈19%. (**d**,**e**) Atomic density in time-of-flight for BECs coupled to the higher modes shown in **a**–**c**, respectively. (**a**,**d**) The nodal structure factor is not apparent for this mode, TEM_10_, which is orthogonal to the TEM_01_ mode shown in Fig. 4l, because for imaging along 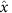, the node in atomic density along 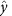 is obscured by the column integration inherent in the absorption imaging process. (**b**,**e**) A nodal structure factor is evident in the first-order Bragg peaks for this even-parity supermode-DW-polariton condensate, because the BEC sits at a cavity nodal plane oriented parallel to the atomic absorption imaging axis 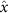. (**c**,**f**) By contrast, no nodal structure factor is evident for this nearly azimuthally symmetric supermode because (1) most atoms are located in the central antinode and (2) the nodal plane parallel to 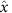 is obscured by the atomic density in the ring parallel to 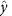. The high-order fringes along 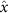 in **b** are, we believe, the admixture into the supermode-DW-polariton condensate of a very high-order transverse mode or set of modes from a family of modes originating from half a free-spectral range lower in frequency. Although such modes are not common, we do find them at very specific detunings Δ_c_ from the cavity modes. The colourbar for cavity emission in **a**–**c** is to the right of **a**. The colourbar for **d**–**f** is to the right of **d**. For all panels, the axes are indicated with labelled arrows in **a**, for the atomic density, and in **d**, for cavity emission. The white scale bars equal **a**, twice the TEM_00_ optical waist 2*w*_0_=70 μm at the BEC; and **d**, the recoil wavevector *k*_r_=2*π*/*λ* with *λ*=780 nm.

Time-of-flight expansion of the atoms yields the atomic momentum distribution and the lattice defect appears as a node in the (*k*_*y*_, *k*_*z*_)=(±1, ±1)*k*_r_ Bragg peaks of the expanding BEC's interference pattern; see simulation in Fig. 4g. The node may be understood as a structure factor resulting from the low-momentum modulation of the organized atomic wavefunction caused by coupling to the transverse nodal structure of the supermode. The observability of a structure factor in the momentum distribution of BECs organized in orthogonally oriented and higher-order modes is shown in Fig. 5.

**Figure 6:**
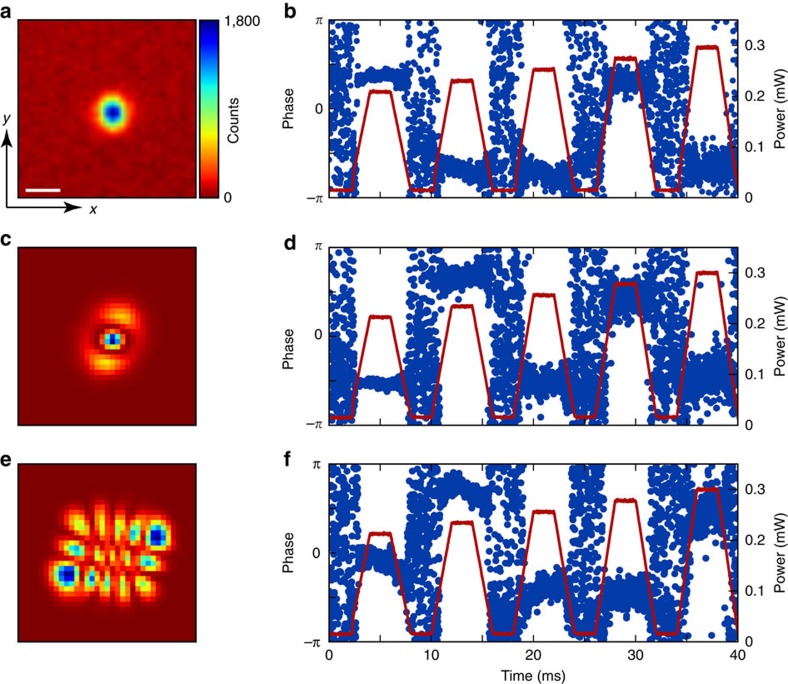
Heterodyne measurement of the phase locking of the supermode-DW-polariton condensate. (**a**,**c**,**e**) Images of the photonic component of the supermode-DW-polariton condensates for which phase measurements are made. The supermode in **a** is TEM_00_, that in **c** is a mixture of *α*≈81% TEM_02_ and *β*≈19% TEM_20_, whereas the supermode in **e** is dominated by the TEM_42_ mode. (**b**,**d**,**f**) Heterodyne measurements of the phase of the supermode-DW-polariton condensates in **a**,**c**,**e**. The red overlay shows the power of the transverse pump versus time as the atoms self-organize and melt five times in the single experimental cycle. The supermode-DW-polariton condensate picks one of two possible phases, separated by ∼*π*, with respect to the transverse pump for each organization event. This is a signature of the *Z*_2_ symmetry being broken on condensation: the locking of phase corresponds to the two possible atomic checkerboard patterns^21^,^22^,^30^. (**c**–**f**) This phase locking is evident even for supermode-DW-polariton condensates with higher-order superpositions of the cavity supermodes. The colour field for cavity emission in **a**,**c**,**e** is to the right of **a**. For **a**,**c**,**e**, the axes are indicated with labelled arrows in **e**. The white scale bar in **e** equals twice the TEM_00_ optical waist 2*w*_0_=70 μm at the BEC.

Stronger pumping modifies the matter wavefunction by increasing the light–matter coupling nonlinearity, as may be seen by the emerging node at the centre of the zeroth-order and (±2, 0) Bragg peaks in Fig. 4k. This distortion of the condensate wavefunction is similar to that which happens in dilute gas BECs on increasing interaction energy. Lastly, we note that these supermode-DW-polariton condensates are observed to break the same *Z*_2_ symmetry observed in single-mode DW-polariton condensates^21^,^22^,^30^. Figure 6 presents measurements of pump-cavity field phase locking.

## Discussion

Our results demonstrate it is possible to study the DW polariton condensation phase transition when there are a few degenerate (or nearly degenerate) cavity modes. We have shown this has notable effects on properties such as the structure factor in the time-of-flight image of atomic density. The demonstration of this phase in the few-mode-degenerate system paves the way for measurements of critical behaviour in non-equilibrium quantum systems employing fully multimode cavity quantum electrodynamics. The multimode regime can be easily realized in our existing apparatus by tuning the cavity mirror spacing to confocality *in situ*. Indeed, this capability has already been achieved in our system in a robust manner, as discussed in ref. 31. To explore critical behaviour in phase transitions of open quantum systems, the crucial requirement is the existence of a continuum of modes, so that the response of the system at different length and timescales is not all dependent on the same few modes. The number of modes should be large enough that the beyond-mean-field corrections to the threshold power are greater than the ability to measure and control this threshold power.

Multimode cavity quantum electrodynamics, in the limit of multimode collective ultra-strong coupling wherein the collective coupling is larger than the bandwidth of the degenerate modes, strongly mixing them, should provide access to a much more exotic condensation transition. This fluctuation-induced first-order Brazovskii transition is predicted to yield a superfluid smectic-like quantum liquid crystalline order of the intracavity BEC^23^. This opens avenues to study the interplay of quantum liquid crystallinity and unconventional superfluidity under controlled dimensionality and disorder, as well as the study of superfluid glasses and spin glasses^23^,^32^, longstanding problems in statistical mechanics.

## Methods

### Apparatus

We prepare a nearly pure ^87^Rb BEC of 3 × 10^5^ atoms at the centre of our cavity, confined in a crossed ODT with trap frequencies [*ω*_*x*_, *ω*_*y*_, *ω*_*z*_]=2*π* × [59.1(6), 88.0(9), 89.4(5)] Hz. The atoms are prepared in the 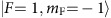 state and a 1.4 G magnetic field is oriented along the *z* axis. The Thomas–Fermi radii of the BEC [*R*_*x*_, *R*_*y*_, *R*_*z*_]=[9.8(3), 8.3(2), 8.3(2)] μm are significantly smaller than the 35 μm waist (1/*e* radius of the cavity field) of the TEM_00_ cavity mode. The crossed ODT is formed by a pair of 1,064 nm laser beams with waists 39 and 20 μm intersecting at 45° in the *xy* plane. Acousto-optic modulators are used to stabilize the intensity of each ODT beam and control its position, allowing us to translate the BEC inside the cavity to control its overlap with the cavity modes.

The cavity is operated in a near-confocal regime in which the length *L* is set to differ from the radius-of-curvature by 50 μm (ref. 31). The *L*=1 cm-long cavity has a free-spectral range of 15 GHz and a single-atom TEM_00_ cooperativity of 2.5: *g*_0_=2*π* × 1.04 MHz and *κ*=2*π* × 132 kHz. A weak 1,560 nm laser is used to stabilize the cavity length using the Pound–Drever–Hall method. In addition, light from this laser is amplified and doubled to generate 780 nm light for the transverse pump and longitudinal probe beams. The wavelength of the locking laser is chosen to achieve a large atomic detuning of Δ_a_=−102 GHz between the pump and the 6 MHz-wide D2 line of ^87^Rb. An electro-optic modulator placed in the path of the locking laser beam allows us to tune the detuning Δ_c_ between the cavity modes and the pump or probe beams. The pump beam is polarized along *x* and is focused down to a waist of 80 μm at the BEC and retro-reflected to create an optical lattice oriented along *y*. At the cavity mirror, the TEM_00_ beam has a waist of 50 μm, which is much smaller than the mirror size. We have checked elsewhere^31^ that this remains true up to modes of order *l*, *m*≃50, and that these modes also exhibit high finesse. This provides an ultimate limit of around 1,250 on the number of transverse modes that can be made resonant exactly at the confocal point.

The cavity modes can be probed using a large, nearly flat longitudinal probe beam propagating along the axis of the cavity. This probe couples to all transverse modes of the low-order families. Reference 31 describes the design and vibration isolation of the length-adjustable cavity.

### Measurements

The cavity output can be directed to three different detection channels. A single-photon counting module can record photon numbers via a multimode fibre coupled to the multimode cavity output. We measure a detection efficiency—from cavity output mirror to detector, including quantum efficiency and losses—of 10% for the low-order modes discussed here. The dispersive shift data in Fig. 2a is taken for an intracavity photon number much less than one and the same Δ_a_ as for the rest of the data, −102 GHz.

**Figure 7:**
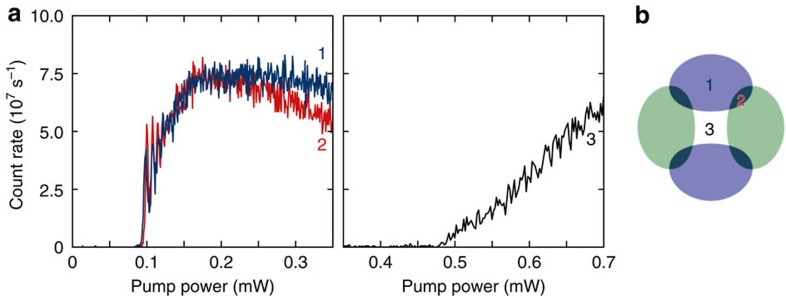
Threshold versus position. (**a**) Cavity transmission, measured on a single photon counter, versus transverse pump power for three positions in the transverse plane of the cavity. Measurement at detuning Δ_c_=−28.8 MHz from the TEM_01_ mode. The three positions are shown in **b**: 1 (dark blue) at the antinode of TEM_10_, 2 (red) in between modes and 3 (black) at the node at centre of the cavity. Onset of superradiance occurs at the same threshold for positions 1 and 2, whereas threshold for position 3 is much less sharp and at higher pump power, illustrating the poorer coupling when the BEC is at a node.

**Figure 8:**
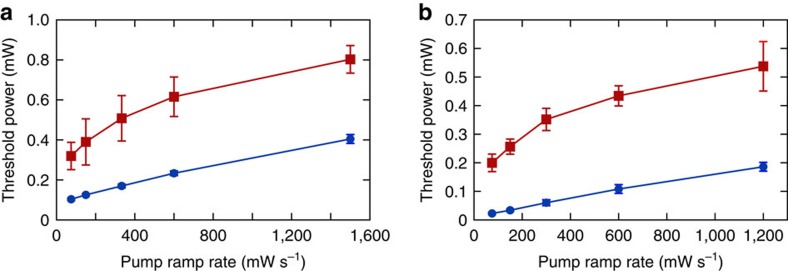
Threshold as a function of transverse pump power ramp rate. (**a**) A comparison between the thresholds for the *l*+*m*=0 (blue) and *l*+*m*=1 (red) modes for a BEC placed at the centre of the cavity. In both cases the BEC is pumped at a large detuning of Δ_c_=−30 MHz. The *l*+*m*=0 mode has an antinode at the centre of the cavity, whereas the *l*+*m*=1 mode has a node. The overall higher threshold of the odd mode is indicative of the worse overlap between the atomic wavefunction and the node of the optical mode. The greater rate of threshold increase with pump power ramp rate indicates that the atoms need more time to adapt to the sign-flip of the odd mode than can be provided during the timescale of the faster ramp. Higher threshold pump power is needed to compensate. A consequence of this can be seen in the slow turn-on of superradiance in the data of Fig. 7b. This dynamical effect hints that motion of the atoms following the turn-on of the cavity light may in some cases lead to effects beyond the linear stability analysis presented in the Methods section, which produced the green curve in Fig. 2m. (**b**) An analogous behaviour is seen in the *l*+*m*=2 family, for the TEM_02_ (blue) and TEM_11_ (red) modes. The former has an antinode at the cavity centre, whereas the latter, although still an even-parity mode, has a node at the cavity centre. The thresholds were measured by pumping at a detuning Δ_c_=−2 MHz from the respective cavity mode. Lines are guides to the eye. Error bars represent 1 s.e.

Superradiant emission from the cavity is observed by monitoring the cavity output on the single-photon counting module. A sharp rise in intracavity photon number as the pump power is increased heralds the condensation transition. See Figs 7 and 8.

**Figure 9:**
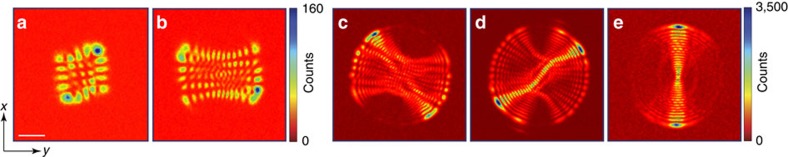
Gallery of supermode-DW-polariton condensates. (**a**–**e**) Photonic components of various high-order supermode-DW-polariton condensates. Emission clipped at large radius by limited aperture of camera imaging optics at this magnification. The colourbar for cavity emission in **a**–**e** is to the right of **b**,**e**. For all **a**–**e**, the axes and scale are indicated with the labeled arrows and the white bar as in **a**, where the bar equals twice TEM_00_ the optical waist 2*w*_0_=70 μm at the BEC.

Alternatively, we can image the emission using an electron-multiplying CCD (charge-coupled device) camera, to spatially resolve the transverse mode content of the cavity emission, although with no temporal resolution. All images of cavity emission are taken in a single experimental run (no averaging of shots with different BEC realizations) and with a camera integration time between 1 and 3.3 ms. The lower signal-to-noise in the images of Fig. 2c–g versus Fig. 2h–l is due to lower intracavity photon number. The images in Fig. 2b with no atoms present are taken with intracavity photon number well above unity. The pump power in Fig. 4k is 70% larger than in Fig. 4j. See Fig. 9 for images of higher-order modes.

The phase difference between the cavity output and pump beam can be determined by performing a heterodyne measurement with a local oscillator beam^22^. The data in Fig. 6 has a frequency offset between pump and signal beams of 11 MHz.

We calibrate the Rabi frequency of the transverse pump by measuring the depth of the pump lattice through Kapitza–Dirac diffraction of the BEC. With the cavity modes detuned far-off resonance, we pulse the pump lattice onto the BEC for a time Δ*t*. The BEC is then released from the trap and the population *P*_m_(Δ*t*) of the diffracted orders at momenta 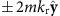 is measured after a time-of-flight expansion. By fitting the measured *P*_m_(Δ*t*) to theory^33^, we extract a lattice depth *V*_0_=Ω^2^/Δ_a_, which allows us to determine the Rabi frequency Ω of the pump.

The momentum distribution of the ^87^Rb cloud is measured by releasing the cloud from the trap and performing resonant absorption imaging after an expansion time *t*_TOF_=17 ms. The appearance of Bragg peaks at |**k**|=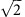
*k*_r_ in the atomic momentum distribution coincides with the onset of superradiant emission. All atomic time-of-flight images are taken in a single experimental run (no averaging of shots with different BEC realizations). The paired atomic absorption and cavity output images in Fig. 4i,j and in Fig. 5a–f are each taken for the same experimental run. The cavity output image in Fig. 4l was taken for the same experimental run as the atomic absorption image in Fig. 4k.

We use the position dependence of the *l*+*m*=1 supermode-DW-polariton condensates (see Fig. 3) to place our BEC at the centre of the cavity modes. The BEC is translated in the *xy* plane using the acousto-optic modulators of the two ODT beams. By monitoring the orientation of the superradiant emission as a function of BEC position, we are able to infer the displacement between the BEC and cavity centre. Exploiting these effects allows us to position the BEC at the cavity centre in both *x* and *y* directions to within 4 μm.

### Mode decomposition

We analyse the transverse mode content of the polaritons in the *l*+*m*=*s* family by decomposing the cavity field into a superposition of unit-normalized, Hermite–Gaussian modes Φ_*lm*_(*x*, *y*; *w*_0_, *x*_0_, *y*_0_). This is achieved by fitting the EMCCD image *I*(*x*, *y*) to the function


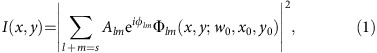


with the mode magnitudes *A*_*lm*_ and phases *φ*_*lm*_ determined as fit parameters. The waist *w*_0_ and centre positions (*x*_0_, *y*_0_) of Hermite–Gaussians are determined from an image of the TEM_00_ cavity mode and are held fixed during the fit. Fixing the phase of the Φ_*s*0_ modes to *φ*_*s*0_=0 allows the fitting algorithm to converge to a local optimum. Using the fitted values of *A*_*lm*_, we extract admixture fractions


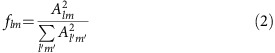


of the Φ_*lm*_ mode in the cavity output.

### Model Hamiltonian

We model the coupled dynamics of the atomic wavefunction 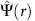 and cavity modes 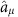 (where *μ*=(*l*, *m*)) with the Hamiltonian


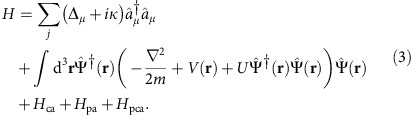


The first term represents the evolution of the cavity modes and second is the familiar Gross–Pitaevskii Hamiltonian for a weakly interacting BEC trapped in a harmonic potential *V*(**r**). The atomic contact interaction is accounted for in the term proportional to *U*. In the regime of large atomic detuning Δ_a_, we can neglect the excited electronic state of the atom, and the atom-light interactions may be described solely through the dispersive light shifts. The cavity–atom interaction becomes


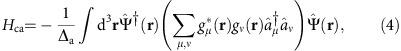


where *g*_*ν*_(**r**)=*g*_0_Φ_*ν*_(***r***)/Φ_00_(0) is the spatially dependent, single-photon (vacuum) Rabi frequency for the cavity mode *ν*. Similarly, for a pump field with a Rabi frequency Ω(**r**), the atom–pump interaction is


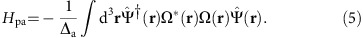


The last term of equation (3) represents the light shift arising from the interference between the cavity and pump fields and is written as


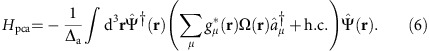


### Simulation of supermode composition at threshold

To predict the location of threshold, and the nature of the supermode at that point, one may make use of a linear stability analysis, assuming a small occupation of the cavity modes and atomic DW excitation^34^,^35^. For the cavity mode, this is straightforward. For the atoms, this corresponds to assuming a condensate wavefunction


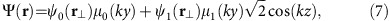


where *μ*_*n*_(*θ*) are 2*π* periodic eigenfunctions of the Mathieu equation, with eigenvalue *a*_*n*_, that is,


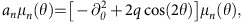


where *q*=−*E*_Ω_/*ω*_r_ and *E*_Ω_=Ω^2^/Δ_a_. These Mathieu functions describe the effects of the pump beam in the *x* direction and do not assume a weak pump lattice. In the cavity direction, *z*, the lattice is assumed weak and so we only consider the first two modes, that is, 1 and 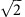
cos(*kx*). As the above expression encapsulates all effects of the longitudinal coordinate, we will suppress the label ⊥ on the transverse coordinates.

We must then solve coupled equations for the atomic transverse envelope functions 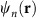 and cavity mode amplitudes *α*_*μ*_. To leading order in perturbation theory, the ground state envelope 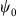 does not change and so corresponds to the solution of the Gross–Pitaevskii equation:


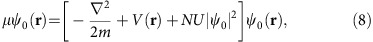


where *μ* is the chemical potential and *N* the number of atoms.

Mean-field equations of motion for the 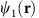 and 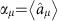 are derived from the Hamiltonian in equation (3),


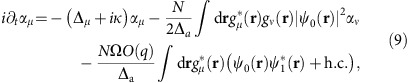



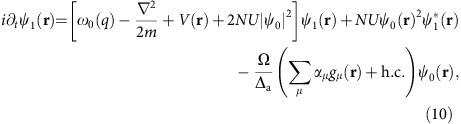


where we have taken Ω(**r**)=Ω cos(*kx*). The spatial dependence of the pump enters through the overlap 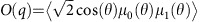, of the first two Mathieu functions due to the cross pump–cavity light field potential. The energy scale *ω*_0_(*q*)=*ω*_r_(1+*a*_1_(*q*)−*a*_0_(*q*)) corresponds to the effective recoil in pump and cavity directions, allowing for the possibility of a deep pump lattice. For a shallow lattice these functions become 1 and 2*ω*_r_, respectively.

From these linearized equations, we may then determine when supermode-DW-polariton condensation occurs, by identifying the point at which the linearized fluctuations become unstable. There is some subtlety to this point, discussed further below. Calculating the growth/decay rates of linearized fluctuations is straightforward, corresponding to an eigenvalue equation. As there are anomalous coupling terms (that is, as *α*_*μ*_ depends on both 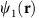 and 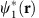, and vice versa) one must use the Bogoliubov–de Gennes parametrization, that is, write 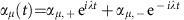 and similarly for 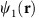. It is convenient to resolve the function 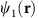 onto some set of basis states. We use the harmonic oscillator basis states, giving a particularly simple result in the limit *U*→0.

With the basis noted above, the eigenvalue problem is given by Det[*A*−*λ*1]=0 where the matrix *A* can be written in the block form in terms of 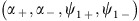 blocks:


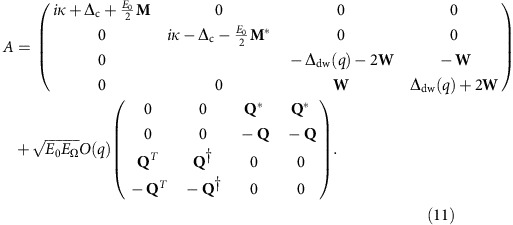


In this expression, the various block matrices are as follows: the matrix **Δ**_c_ is a diagonal matrix consisting of the detuning between the pump laser and each cavity mode. **Δ**_dw_(*q*) is similarly a diagonal matrix describing the energy difference between a given atomic transverse mode function and the ground state mode function. This is a function of *q* as it also includes the energy *ω*_0_(*q*) associated with the different scattering states. The matrices **W** denote the effect of atom-atom interactions, corresponding to the overlap between two atomic modes and the atomic ground state density. That is, they describe scattering off the condensate causing transitions between modes. The matrices **M** denote the dielectric shift due to the atoms, corresponding to the overlap between two cavity modes and the atomic ground state density. (The matrices **M** and **W** differ in general because the cavity beam waist does not match the harmonic oscillator length of the atoms.) The matrix **Q** denotes atom-cavity scattering and involves the overlap of the atomic ground-state mode function with a given excited mode and a given cavity mode. The energy scale is 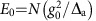 for cavity beam waist *w*.

The condition for the matrix *A* having unstable eigenvalues can be directly related to the idea of non-equilibrium condensation of polaritons^36^. As discussed there, one may directly relate the real frequencies at which the real and imaginary parts of the inverse retarded Green's function vanish to the polariton energies and the effective chemical potential. At the point of condensation, these frequencies meet; that is, there exists a real frequency at which the inverse Green's function vanishes. This point is equivalent to the boundary between the Green's function having unstable and stable poles. For the single-mode cavity, this connection to condensation has been discussed extensively^24^,^34^, including the identification of the low energy effective temperature emerging at the transition. The Green's function which defines all these quantities is directly related to equation (11), although conventional definitions of Green's functions introduce minus signs in alternate rows and columns.

On solving equation (11), one finds that although there is a threshold for instability when **Δ**_c_<0 of nearby modes, there is always an unstable eigenvalue as soon as **Δ**_c_>0 for any mode (that is, the pump is blue-detuned of any mode). However, the growth rate of this instability varies widely with parameters. At low pump powers, the timescale for growth is very long (that is, seconds)^35^. As pump strength increases, there is a sharp threshold where two eigenvalues of equation (11) cross, demarcating a transition to a state that rapidly orders (timescale of microseconds). The curves in Fig. 2 correspond to finding the eigenvector (i.e., mode composition) of this mode which becomes rapidly unstable.

### Structure factor simulation

The calculated density and momentum distributions presented in Fig. 4 are evaluated by numerically integrating the mean-field equations of motion in three dimensions for the atomic wavefunction and cavity mode. Using the Hamiltonian in equation (3), we derive equations of motion for the atomic wavefunction and cavity field under a mean-field approximation where 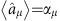 and 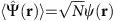. This gives us the coupled differential equations,


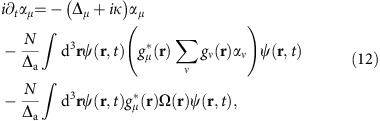



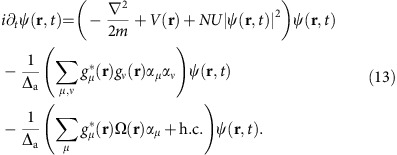


Furthermore, we adiabatically eliminate the cavity field *α*_*μ*_ under the assumption that it equilibrates on a timescale much faster than the atomic motion. To simulate the behaviour presented in Fig. 4, we restrict *α*_*μ*_ to a single mode, either TEM_00_ or TEM_10_, and numerically integrate the equations of motion. The initial atomic wavefunction is set to the Thomas–Fermi distribution associated with our ODT parameters and the initial cavity field is set to *α*_*μ*_(0)=0. The strength of the pump field is increased linearly in time from 0 to Ω(**r**, *t*) to simulate the transverse pumping of our cavity. The *in situ* density distributions shown in Fig. 4c–e are 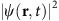. The momentum distributions are obtained by a Fourier transform of the *in situ* atomic wavefunction.

### Condensation of supermode DW polaritons

The model described above is specific to the case of condensation of supermode DW polaritons occurring on top of an existing BEC of atoms, as was studied experimentally. The phase transition to macroscopic occupation of cavity modes can however happen for both Bose condensed and thermal atoms^22^. Indeed, for a single-mode cavity, the distinction of the phase transitions for BEC of atoms and superradiance was discussed by Piazza *et al*.^37^, leading to a phase diagram where either phase can occur independently of the other. However, the signatures we study, such as the appearance of structure in the Bragg peaks from the time-of-flight image, would no longer be visible in the absence of atomic coherence^18^,^19^, that is, only for a coherent atomic state is the momentum distribution direction related by Fourier transformation to the density profile in real space.

The relation of the DW-polariton condensation transition to the coherence of atoms is complicated by the interpretation of single-mode DW-polariton condensation^12^ in terms of the Dicke model. This interpretation is based on the similarity to the proposed realisation of the Dicke model^38^ using two-photon transitions between hyperfine states of the atoms, later realized by Baden *et al*.^15^ In a single-mode cavity, DW-polariton states can be approximately mapped to this model^12^,^13^,^34^ as long as the atoms are Bose-condensed—in this case, two macroscopically occupied momentum states of the atoms play the rôle of the internal states in the proposal^38^. For a non-condensed cloud^37^, or for multimode cavities, such an approximation does not hold. Indeed, before the experiments on a condensate in a single-mode cavity, supermode-DW-polariton condensation in a multimode cavity had been discussed by Gopalakrishnan *et al*.^23^,^39^

### Data availability

Parts of the research data supporting this publication can be accessed from ( http://dx.doi.org/10.17630/eddbeb14-9bab-4058-8033-cc4672770e0c). The remaining data are available from the corresponding author on reasonable request.

## Additional information

**How to cite this article:** Kollár, A. J. *et al*. Supermode-density-wave-polariton condensation with a Bose–Einstein condensate in a multimode cavity. *Nat. Commun.* **8,** 14386 doi: 10.1038/ncomms14386 (2017).

**Publisher's note**: Springer Nature remains neutral with regard to jurisdictional claims in published maps and institutional affiliations.
